# The Structure, Oxidation States, and Energetics of Co Nanoparticles on CeO_2_(111): An STM and DFT Study

**DOI:** 10.1021/acs.jpcc.4c03911

**Published:** 2024-08-28

**Authors:** Md. Saeedur Rahman, Nishan Paudyal, Linze Du Hill, Jing Zhou, Ye Xu

**Affiliations:** † Cain Department of Chemical Engineering, Louisiana State University, Baton Rouge, Louisiana 70803, United States; ‡ Department of Chemistry, University of Wyoming, Laramie, Wyoming 82071, United States

## Abstract

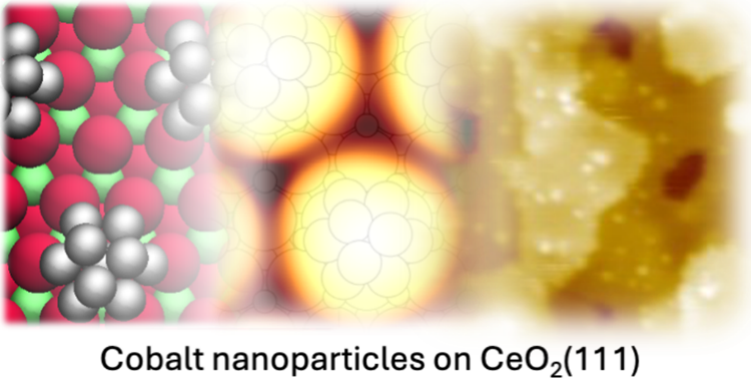

Co nanoparticles (NPs) dispersed on ceria have been widely studied as active catalytic materials for many industrially relevant reactions. The detailed nature of such particles and the factors affecting their interaction with ceria remain to be better understood. In this study, a very low coverage (∼0.02 ML) of Co is deposited on a model CeO_2_(111) thin-film surface and is examined using scanning tunneling microscopy (STM) and X-ray photoelectron spectroscopy (XPS). The Co NPs that nucleate on terrace sites grow with coverage in this range to a maximum size of ca. 40 Co atoms, with an average diameter and height of 16.1 and 1.1 Å, respectively. Global minimization of the structures of Co NPs consisting of up to 23 Co atoms on CeO_2_(111) is performed based on the minima hopping algorithm and density functional theory (DFT) calculations, and the energetic and chemical properties of the resulting NPs are analyzed. While the theoretical findings are consistent with the STM observations on the strong Co-ceria interactions and the prevalence of oxidic Co species, some notable discrepancies are identified, including inconsistent aspect ratios and the existence of a low oxidation state Co^δ+^ species. The combined experimental and theoretical findings provide new insights into Co NPs formed on ceria and identify areas requiring further investigation.

## Introduction

Co nanoparticles (NPs) dispersed over ceria have been studied for important catalytic reactions including dry reforming of methane,^1,2^ Fischer–Tropsch reaction, steam reforming of ethanol,^3−6^ CO_2_ hydrogenation reaction,^7−9^ CO oxidation reaction,^10^ water gas shift reaction,^11,12^ and oxidation of soot particulates.^13,14^ Ceria exhibits unique redox properties and oxygen storage capacity.^15−17^ As an oxide support of metals such as Co, ceria can affect their dispersion, size, morphology, and electronic properties, and thus influence their catalytic reactivity. It has been shown that Co supported on ceria delivers interesting catalytic activity that can be attributed to strong metal–support interaction (SMSI).^2^ For instance, ceria can modify the electronic properties and oxophilicity of Co. Both metallic and oxidized forms of Co can form on ceria, and they can play different roles in a given chemical reaction, making chemical properties of Co NPs on ceria very different from those of bulk-like cobalt particles.^2,18,19^

To elucidate the nature of the surface chemistry of ceria-supported Co catalysts, it is important to gain a fundamental understanding of the growth, chemical state, and energetics of Co NPs over ceria. Previously, the growth of Co over well-defined CeO_2_(111) surfaces has been studied experimentally using X-ray photoelectron spectroscopy (XPS), scanning tunneling microscopy (STM), and low-energy ion scattering (LEIS) techniques under ultrahigh-vacuum (UHV) conditions.^20−22^ Among the crystalline facets of ceria, the CeO_2_(111) facet is of main interest as the model support surface for Co because of its high stability.^17^ In general, there is an agreement among XPS studies that demonstrate the oxidation of Co to Co^2+^ occurs at low Co metal coverages on CeO_2_ that is accompanied by the reduction of Ce^4+^ cations to Ce^3+^ in ceria. This process is believed to take place at the Co-ceria interface.^20−22^ The metallic Co appears upon increasing Co coverage to ∼0.5 monolayer (ML) and both metallic and +2 states of Co are present on CeO_2_.^21,22^ Further increase of the Co coverage increases the amount ratio of metallic Co to Co^2+^ species. XPS and LEIS studies have also suggested that there is a transition from the growth of two-dimensional (2D) to three-dimensional (3D) Co NPs on CeO_2_(111) with the increase of Co coverages at room temperature.^20^ Our recent STM study of Co on CeO_2_(111) confirmed the formation of small 2D Co NPs at 300 K at low coverages (e.g., 0.02 ML). These NPs are uniformly distributed on the ceria surface, suggesting a strong metal–support interaction between Co and CeO_2_. Increasing Co coverage from 0.02 to 0.10 ML predominantly increases the number of Co NPs on CeO_2_(111). Further increase of Co coverage causes an extensive growth in size to form 3D NPs. The height of Co NPs increases from 1.6 ± 0.3 Å at 0.1 ML Co to 2.0 ± 0.6 Å at 0.2 ML Co coverage and 3.3 ± 1.0 Å at 0.4 ML Co.^22^ To follow up with that study, we now investigated the Co growth at a very low Co coverage (∼0.02 ML), which allows for the detailed atomic-level understanding of the nucleation and growth of Co as well as the elucidation of the interaction between Co and CeO_2_. In this study, 0.02 ML of Co deposited on a model CeO_2_(111) surface was examined using STM and the sizes of Co NPs were quantitatively analyzed. The experimental results are complemented with density functional theory (DFT) calculations, which provide information on the energetics, structure, and oxidation state of Co NPs consisting of up to 23 Co atoms on CeO_2_(111). The combined experimental and theoretical findings provide insight into the structural and chemical properties of Co NPs formed on the terrace sites of ceria.

## Methods

### Growth and Characterization of CeO_2_(111) Thin Films and Co/CeO_2_(111)

The growth and characterization of Co over CeO_2_(111) thin films were carried out using a UHV surface analysis system manufactured by Omicron Nanotechnology with a base pressure below 5 × 10^–11^ Torr. The instrument consists of a variable-temperature scanning tunneling microscope (VT-STM XA 650), a DAR 400 twin anode X-ray source with an EA 125 U1 hemispherical electron analyzer, a four grid SPECTALEED optics, an ISE 5 cold cathode sputtering ion source, single- and triple-pocket electron beam evaporation sources, a sample manipulator with electron beam heating capabilities, and a fast-entry load lock as described elsewhere.^22,23^

A Ru single crystal (Princeton Scientific Corp, 10 mm in diameter, one side polished, roughness <0.03 μm, orientation accuracy <0.1°) was used as the substrate for the growth of CeO_2_(111) thin films. The Ru surface was cleaned via Ar ion sputtering (1 keV, ∼ 3 μA sample current), followed by annealing at 1300 K for 45 s. The surface cleanliness and long-range order were confirmed through STM, XPS, and low-energy electron diffraction (LEED) studies. Fully oxidized CeO_2_ thin films were prepared by evaporation of cerium (Alfa Aesar, 99.9% pure) onto the Ru (0001) surface at 700 K in oxygen with a chamber pressure of 2 × 10^–7^ Torr.^24−26^ The film was subsequently annealed at 1100 K in the presence of oxygen to make it well ordered. STM results show that the film consists of approximately 5–6 O–Ce–O trilayers that fully cover the Ru substrate. The step height of each trilayer is measured to be 3 Å, which is consistent with the 3.1 Å spacing of O–Ce–O in the CeO_2_(111) fluorite bulk structure.^17^ Co (Alfa Aesar, 99.995%) was deposited over CeO_2_(111) thin films at room temperature as described in our previous study.^22^

The surface morphology of as-prepared Co/CeO_2_(111) was studied with STM. All STM images were taken using a tungsten tip at room temperature in the constant current mode with the current set point of 0.2 nA and sample voltage of +3 V. The Scanning Probe Image Processor (SPIP) software was used to measure the diameter and height of individual Co NP within a single STM image. The particle density was determined based on the measurement of the number of NPs counted over the selected image. Co coverage was estimated based on the mean particle size and density as demonstrated in STM studies of Co over CeO_2_(111) by our group as well as Co over TiO_2_(110) by Chen and co-workers.^22,27^ One monolayer coverage of Co is referenced to the packing density of the Co(0001) plane that is 1.8 × 10^15^ atoms/cm^2^. The chemical state of Co NPs formed upon deposition over CeO_2_(111) at 300 K was examined using XPS. High-resolution XPS scans of Ce 3d, Co 2p, and O 1s were collected with a 0.020 eV step and averaged over two sweeps. All XPS spectra were taken using a Mg Kα radiation source (1253.6 eV, 15 kV, 20 mA) with a fixed electron passing energy of 50 eV and an entrance slit size of 6 × 12 mm^2^.

### Theoretical Methods

Periodic spin-polarized DFT calculations were performed in the generalized gradient approximation (GGA) using the Perdew, Burke, and Ernzerhof exchange-correlation functional^28^ as implemented in Vienna Ab initio Simulation Package (VASP).^29^ The Projector Augmented Wave (PAW) method^30^ was used to represent the potentials due to the nuclei and core electrons of the elements involved, including Co(3d4s), Ce(4f5d6s), and O(2s2p).

The models for the CeO_2_(111) surface were slabs consisting of several O–Ce–O trilayers in the z direction with either a (3 × 3) or (4 × 4) surface unit cell. (3 × 3) surface unit cells were used to model Co_*x*_ with *x* ≤ 10, while (4 × 4) surface unit cells were used to model larger Co_*x*_ with 10 < *x* ≤ 23. These unit cell sizes represent a compromise between computational efficiency and a need to model Co NPs at low surface density, which this study focuses on. The periodic slabs were separated by 12 Å of vacuum or more in the z direction. Co atoms were placed on one side of the slab only, with dipole corrections applied in the *z* direction.^31^ The reciprocal space was sampled at the Γ point only.

#### DFT + U

The DFT + U method developed by Dudarev et al.^32^ was used to offset the tendency of LSDA (local spin density approximation) and GGA to delocalize strongly correlated electrons. The inherent self-interaction error prevents the localization of the 4f electrons on Ce atoms that are left behind upon oxygen vacancy formation in ceria.^33^ A small effective U value (≲ 3 eV) has been found by multiple authors, supported by experimental measurements, to give more accurate energetics for chemical reactions,^34−38^ whereas a higher U value of 4–5 eV has been shown to produce a better description of localized 4f electrons on reduced Ce centers.^39−41^ Further, large U values have been applied to the *d* states of bulk transition metal oxides to correct the self-interaction error.^42^

Below we report energetic and structural results obtained using the DFT + U method with U = 3.5 eV applied to the 4f states of Ce, which represents a compromise between the needs for accurate descriptions of energetics and electronic structure. The calculated equilibrium lattice constant of CeO_2_ is 5.494 Å (U = 3.5 eV), which overpredicts the experimental value of 5.41 Å^43^ by 1.6% or less. We also applied a U value to the 3d states of Co, which was determined to be 1.18 eV by matching the calculated cohesive energy of bulk Co metal in the *hcp* phase to the experimental value (4.39 eV). This is because the GGA-PBE cohesive energy without corrections deviates significantly from the experimental value.^44^ We reasoned that an inaccurate description of the bond strengths among Co atoms could bias the structures of Co NPs adsorbed on ceria predicted computationally.

#### Global and Local Geometry Optimization

To globally optimize the geometry of a supported Co NP, the minima hopping (MH) scheme of Goedecker^45^ as implemented in the Atomic Simulation Environment^46^ by Peterson^47^ was used. Each system was initially thermalized to *T*_0_ = 2000 K in a Maxwell–Boltzmann distribution and then allowed to evolve according to ab initio NVE molecular dynamics. The initial *E*_diff_, i.e., the energy acceptance criterion, was set to 1.0 eV. *T*_0_ and *E*_diff_ were continuously and automatically adjusted by the algorithm to sample relevant regions of the potential energy surface while avoiding revisiting known minima. In addition, Hookean constraints implemented by Peterson^47^ were imposed to keep Co atoms from moving more than ca. 6 Å above the top atomic layer of lattice oxygen. Several MH runs were carried out using different initial structures for a given number of Co atoms, *x*. For each *x*, enough iterations were carried out until the accumulative acceptance rate dropped below 25%. To speed up the search, the Kohn–Sham valence states were expanded in a plane wave basis set up to 250 eV, and Co NPs were placed on a slab containing one O–Ce–O trilayer (i.e., 3 atomic layers) with all Co atoms and the top atomic layer of lattice O relaxed. Several lowest energy configurations out of the list of energy minima accepted by the MH algorithm were then optimized on full surface models below.

For further optimization, the Kohn–Sham valence states were expanded in a plane wave basis set up to 400 eV. CeO_2_(111) slabs containing three O–Ce–O trilayers were used, with the topmost O–Ce–O trilayers and all Co atoms adsorbed thereon relaxed, while the bottom two trilayers were fixed at their bulk positions. The spin of each surface structure was fully relaxed during geometry optimization, and several integral spin states nearest the resulting spin were manually checked to determine the lowest-energy spin state. The total magnetic moment of the Co_*x*_ NPs was found to increase linearly with *x* with a slope of 1.91 μ_B_. Geometry optimization was performed until the maximum residual force was 0.03 eV/Å or less for each relaxed degree of freedom.

The average formation energy (Δ*Ẽ*) of a Co NP supported on CeO_2_(111) was calculated using the following relation:
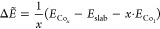
1

Also, the differential adsorption energy (δ*E*) was defined as
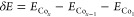
2Here, *E*_Co_*x*__ is the total energy of a surface with *x* Co atoms adsorbed on it, *E*_slab_ is the energy of a clean CeO_2_(111) slab without any Co atom, and *E*_Co_ is the energy of an isolated Co atom in the gas phase. A more negative Δ*Ẽ* or δ*E* corresponds to more exothermic adsorption of Co atoms and therefore indicates greater stability of an NP.

## Results and Discussion

### Size, Dimensions, and Chemical State of Co NPs over on CeO_2_(111) at 300 K by XPS and STM

**Figure 1:**
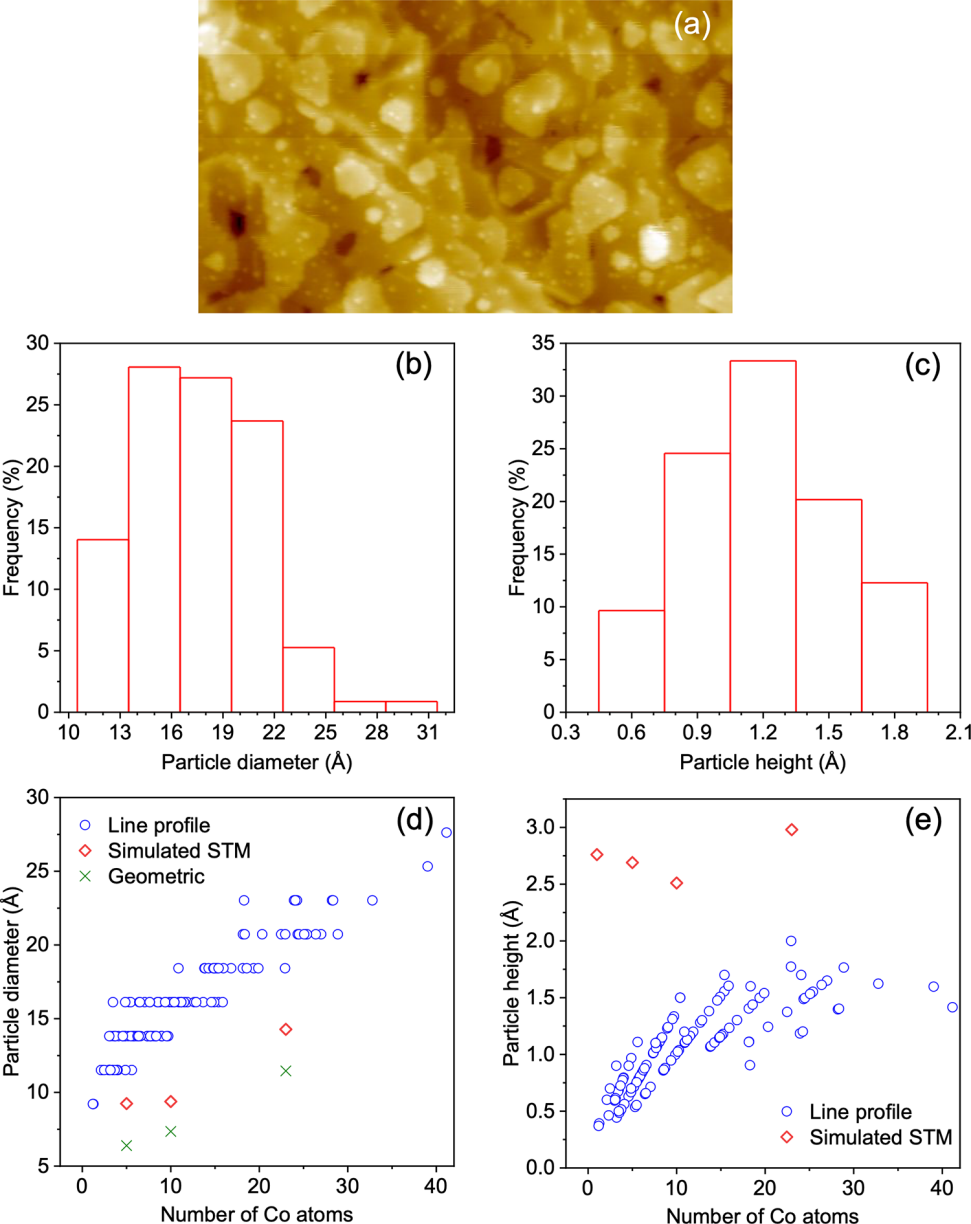
(a) STM image (120 × 70 nm^2^) of ∼0.02 ML Co deposited on CeO_1.97_(111), taken at 0.2 nA set point current and +3 V sample voltage. Histograms for (b) diameter and (c) height of Co NPs on terrace sites in (a). (d) Diameter and (e) height of Co NPs found on terrace sites in (a), based on the line profile analysis plotted against the estimated number of Co atoms in each NP. Apparent diameter and height in simulated STM for several Co NPs modeled and optimized theoretically (see below), and widest (geometric) distance between any pair of O_latt_ atoms bonded to each of these Co NPs, are included for comparison.

Experimentally, we examined Co NPs on CeO_2_(111) with STM closely. Shown in Figure 1a is a representative image of the Co/CeO_1.97_(111) thin-film surface collected after deposition of a very low coverage of Co (e.g., ∼ 0.02 ML) onto CeO_1.97_(111) at room temperature. It is known that CeO_2_ thin films grown under vacuum can contain a small amount of oxygen vacancy defects with associated Ce^3+^ cations.^21^ The reported CeO_2_ films in the study are slightly reduced with ∼6% Ce^3+^ cations and a stoichiometric value of the film of 1.97. The apparent diameter and height of each Co NP within the image of 120 × 70 nm^2^ in Figure 1a were measured using the line profile mode in the SPIP software. Our analysis shows that the particles are quite flat. They are on average 15.9 ± 3.3 Å in diameter and 1.1 ± 0.4 Å in height, with a particle density of 2.7 × 10^12^/cm^2^. Furthermore, the number of NPs on terrace sites is similar to that at the step edges. To better correlate with the DFT results, only information on the NPs on terrace sites is reported in Figure 1b–e.

Most of Co NPs on terrace sites exhibit a diameter between 9.2 and 27.6 Å and a height between 0.4 and 1.7 Å, with the average being 16.1 ± 3.5 and 1.1 ± 0.3 Å, respectively. The fact that Co forms numerous small particles that are uniformly distributed over the CeO_1.97_ surface indicates a strong interaction between Co and ceria and an island nucleation mode. The number of Co atoms in each NP was then determined using the equation below assuming a truncated hemispherical shape for the NPs, and the results were plotted against the NP height and diameter (Figure 1). Here, *d* is the diameter, *h* is the height, and *r*_Co_1__ is the radius of a Co atom in Co metal (a value of 1.35 Å was used in the study^48^). V_NP_ and V_Co_1__ represent the volumes for a Co nanoparticle and a Co atom, respectively.
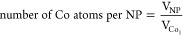
3Here, in the case of *h* being much less than *d*,
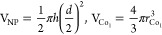
4About 56% of the NPs measured contained 10 Co atoms or fewer, and 85% contained 20 Co atoms or fewer. For Co NPs, an increase in the number of Co atoms from 1 to 40 causes the diameter to increase by about 18.4 Å. However, the height increases by a much smaller value of ca. 1.5 Å.

The estimated number of Co atoms in each NP is approximate due to the assumptions of the particle shape, Co atomic size, as well as tip convolution effects, which can, in particular, lead to an overestimation of the particle diameter. NPs containing four or fewer Co atoms, which can be reasonably concluded to consist of a single atomic layer, exhibit a height mostly between 0.4 and 0.9 Å. It is reasonable to expect there to be a range of values for the atomic layer height for Co measured in STM, considering that the CeO_2_(111) thin-film surface can contain defects. Those NPs containing between 4 and 8 Co atoms show an apparent height between 0.5 and 1.1 Å. Further increasing the number of Co atoms to 10, the NPs mostly show an apparent height within 1.0 and 1.5 Å, corresponding to the Co NPs with two atomic layer high. The NPs consisting of more than 10 Co atoms in Figure 1a mostly exhibit an apparent height between 1.1 and 1.7 Å.

**Figure 2:**
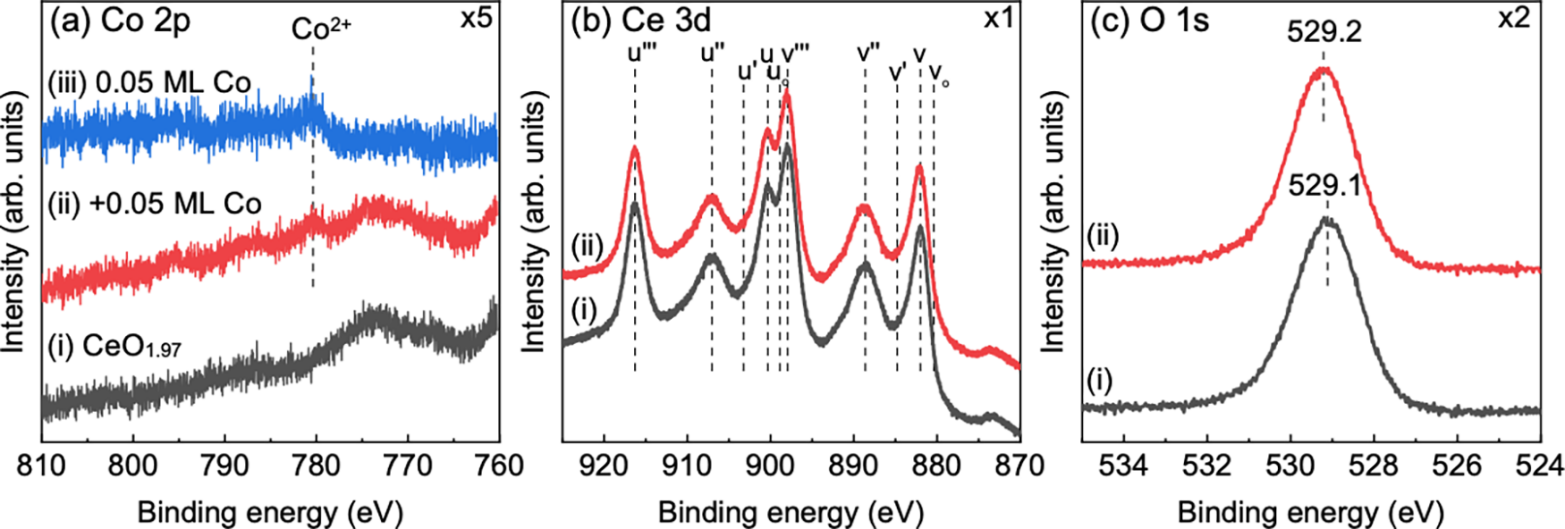
XPS spectra of (a) Co 2p, (b) Ce 3d, and (c) O 1s collected over (i) CeO_1.97_(111) and (ii) CeO_1.97_(111) after Co deposition at 300 K. The spectrum labeled (iii) is the *difference* between Co 2p XPS spectra (i) and (ii), as shown in (a).

The chemical state of Co after deposition over the CeO_1.97_(111) surface at room temperature was studied at slightly higher coverages (∼0.05 ML) using XPS, by monitoring the Co 2p region with a binding energy range between 760 and 810 eV. Within this region, there is a contribution to the signal from the Ce element from the ceria film. Therefore, to obtain the net signal originating from Co, the Co 2p XPS region collected from the pure CeO_1.97_(111) surface was subtracted from that after Co deposition. XPS results (Figure 2) indicated that Co is present in +2 state as evident with the Co 2p_3/2_ peak at 780.4 eV.^20,22^ Compared to the binding energy of 780.0 eV associated with Co^2+^ species in bulk CoO,^49^ there is a shift of 0.4 eV to a higher binding energy value in the Co 2p_3/2_ peak for Co over CeO_1.97_(111), which have been previously attributed to the formation of small particles of Co–O–Ce or CoO,^22^ or may be attributable to a different oxidic Co species. The XPS signal for the characteristic satellite peak at 786.7 eV associated with Co^2+^ is too low to be observed at a 0.05 ML Co coverage. However, it became more pronounced when the Co coverage was increased to 0.2 ML or higher as demonstrated in our previous study.^22^ The oxidation of Co to Co^2+^ is consistent with the reduction of Ce^4+^ cations to Ce^3+^ cations over the CeO_1.97_(111) support. It is known that the Ce 3d XPS region consists of 5 pairs of spin–orbit signals originating from different Ce 4f configurations of Ce^4+^ and Ce^3+^ cations. These peaks are labeled as u and v that correspond to spin–orbit doublets of 3d_3/2_ and 3d_5/2_, respectively (Figure 2b).^23,50−52^ Upon deposition of 0.05 ML Co, there is a reduction in the peak intensity related with Ce^4+^ with a corresponding increase in the peak intensity associated with Ce^3+^ and the Ce^4+^% value decreases by ∼4% based on the analysis of the Ce 3d XPS spectra as described in detail elsewhere.^53,54^ The reduction of the ceria film upon Co deposition is also reflected in the shift of O 1s lattice peak from 529.1 eV toward the higher binding energy of 529.2 eV as shown in Figure 2c, as a result of band bending for a partially reduced ceria.^17,51^

### Nanoparticle Energetics and Structure as a Function of Size

**Figure 3:**
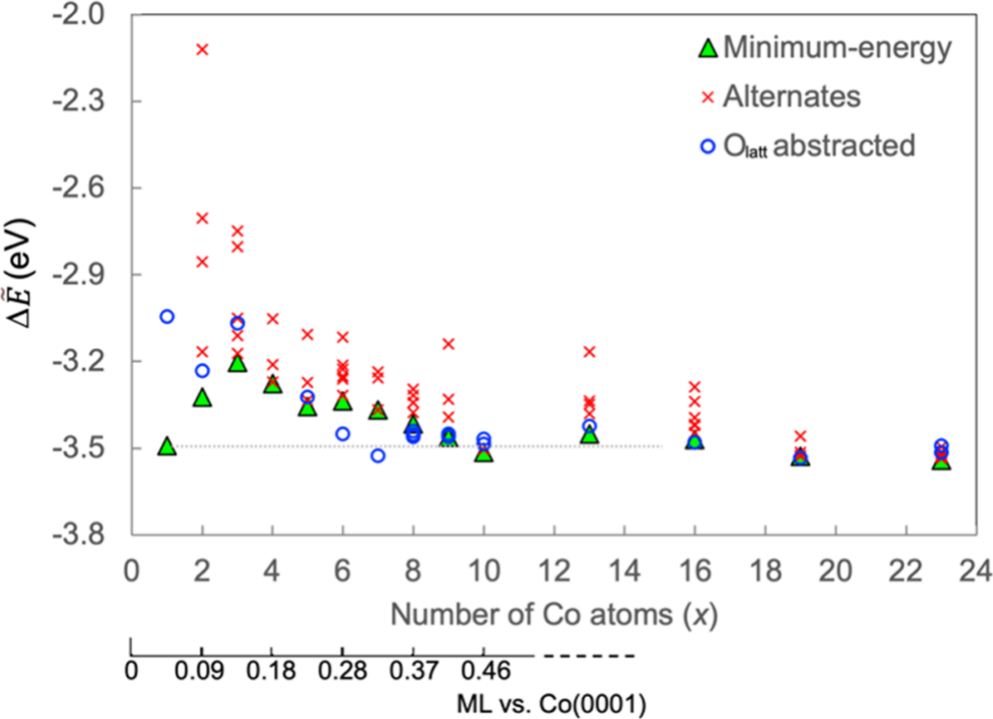
DFT-calculated average formation energy (Δ*Ẽ*) vs number of atoms in Co NPs supported on CeO_2_(111). An equivalent monolayer scale is provided for comparison. Green triangles represent the global minimum-energy Co-only configuration for each size. Alternate, less stable configurations as well as those that contain an O atom extracted from the surface are included for comparison.

**Figure 4:**
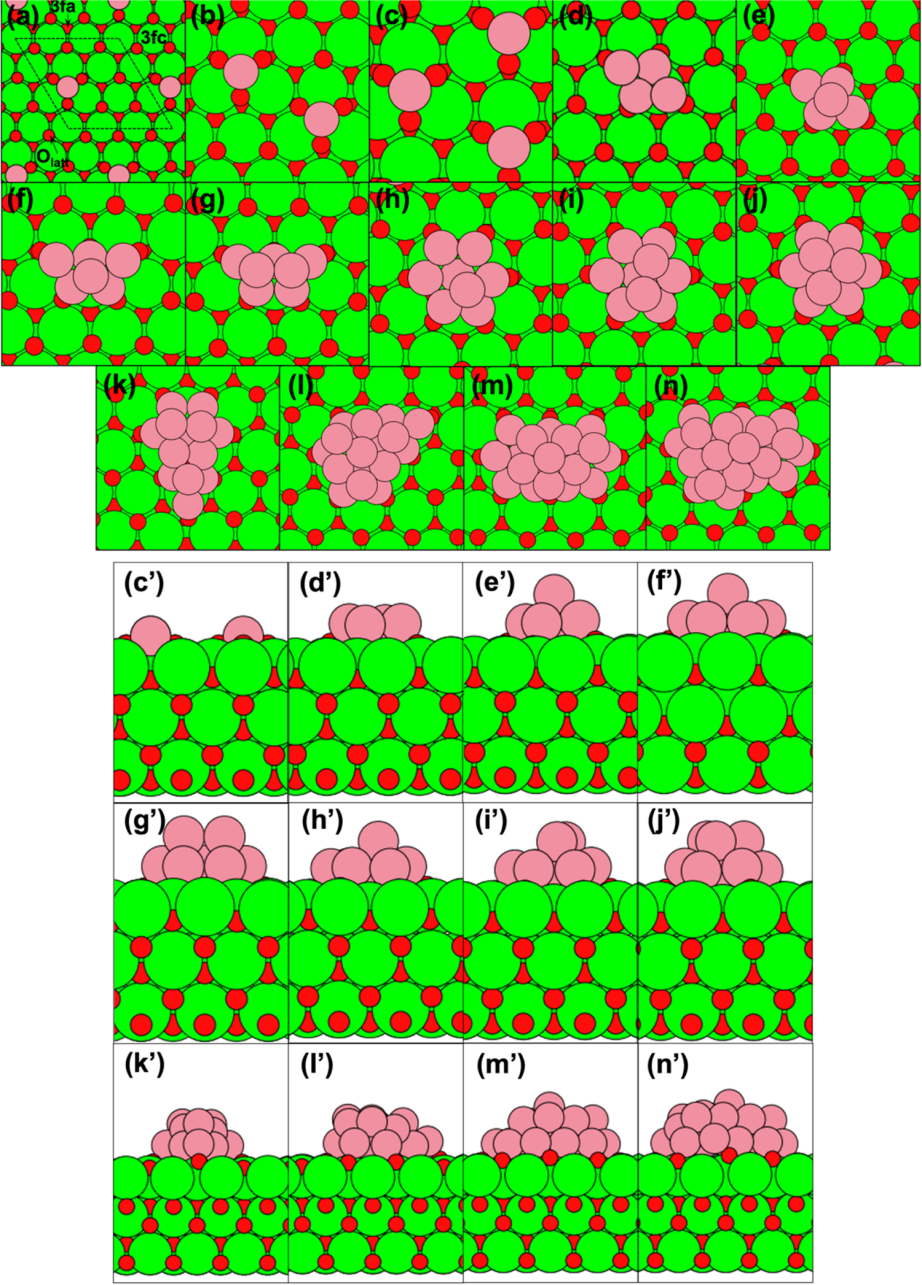
(a–n) Top views of globally energy-minimized structures for Co_*x*_ on CeO_2_(111), *x* = 1–10 and 13, 16, 19, and 23. The (3 × 3) surface unit cell is outlined in (a). Side views for (c-n) are shown as (c′–n′), corresponding to viewing the structures (c–n) in a line of sight directly from the bottom edge to the top edge in each snapshot. Color code: Ce = green, O = red, and Co = pink. For clarity, the Ce cations are shown as larger spheres than the lattice oxygen, and periodic images of clusters are removed in the side views.

When the mobility of NPs is limited or absent, evolution of NPs will occur through the addition or subtraction of single atoms. Also, as the STM results above show, a range of sizes exists even at a very low deposition amount that can be controlled experimentally. Here we use “size” to refer to the number of Co atoms in a Co NP. From the analysis, only ∼15% of the analyzed Co NPs exceeded 20 atoms. Therefore, we explore theoretically how the energetics and structure of Co NPs vary with the number of Co atoms in a NP, *x*, up to 23 atoms. The average formation energy (Δ*Ẽ*) of Co NPs supported on CeO_2_(111) is plotted as a function of *x* in Figure 3. The global minimum-energy (ME) structures that we have determined using the MH approach for each *x* are represented by green triangles, whereas stable but higher-energy structures, which represent less stable local minima on the potential energy surface, are represented by red “×”. Snapshots of the ME structures are shown in Figure 4.

A salient feature of Co adsorption on CeO_2_(111) is that up to three Co adatoms prefer energetically to remain separate when placed in a (3 × 3) surface unit cell. This differs from many metal/oxide deposition systems where the formation of dimers and trimers from adatoms is energetically favorable,^55−58^ which initiates the nucleation process. Upright and flat-lying dimers are notably less stable than two Co adatoms. An upright triangular trimer is much less stable than three adatoms, while a flat-lying triangular trimer is energetically close to, but still less stable than, three adatoms residing on three next-nearest-neighbor 3-fold anion site (3fa) sites, i.e., being as far apart from one another as possible. This characteristic reflects strong Co-ceria interactions, which is stronger than Ni as up to two Ni adatoms prefer to remain separate from one another in a (3 × 3) surface unit cell of CeO_2_(111).^59^

A Co adatom is strongly adsorbed on a 3fa site (Figure 4a) and draws the three nearest O_latt_ atoms closer to it, with Δ*Ẽ* = −3.49 eV for one Co adatom per (3 × 3) surface unit cell. Adsorption on the other high-symmetry sites on CeO_2_(111), including 3-fold cation and lattice oxygen sites (3 fc and O_latt_, Figure 4a), is not stable where a Co adatom spontaneously relaxes into the nearest 3fa site. The attraction of the O_latt_ atoms to a Co adatom represents a form of strain imposed on the oxide lattice by the strong Co–O chemical bonds. This strain, together with the positive charge of the adatom and the formation of Ce^3+^ (discussed later), which are larger than Ce^4+^, causes Co adatoms to repel one another and Δ*Ẽ* to become progressively less negative as *x* increases from 1 to 3. Beyond *x* = 3, Δ*Ẽ* generally becomes more exothermic with NP formation and increasing *x*, but some NP sizes buck the trend, e.g., *x* = 6 and 13, that have slightly less exothermic Δ*Ẽ* than NPs of the adjacent sizes.

All of the ME Co NPs that we have identified possess some degrees of symmetry. The ME structure for four Co atoms is found to be a flat, one-atom-high diamond structure (Figure 4d), with a bilayer tetrahedral cluster being just 0.01 eV less stable. From *x* = 4 to 10, one can discern the pattern in which additional Co atoms are attached to existing NPs to yield a succession of ME structures. For instance, a Co atom is added to the top site of Co_4_ to yield Co_5_ (Figure 4e), following which another Co atom is added to one side of the Co_5_ to yield Co_6_ (Figure 4f), and so on. For Co_5–10_, the most stable flat, one-atom-high structures that we find are all less stable than the ME structures reported in Figure 4 in which Co atoms occupy the second atomic layer. The closest that one- and two-atom-high structures get is for *x* = 7, where a flat hexagonal Co_7_ cluster (not shown) is just 0.01 eV less stable than the ME Co_7_ (Figure 4g). Co_8–10_ (Figure 4h–j) can be viewed as derived from adding 1–3 Co atoms on top of the hexagonal Co_7_. The larger ME Co NPs do not exhibit well-defined packing so that their heights cannot be unambiguously defined in terms of number of layers of Co atoms parallel to the CeO_2_(111) surface, although Co_19_ and Co_23_ are visibly taller than the smaller clusters (Figure 4m′,n′). |Δ*Ẽ*| is expected to approach 4.39 eV/atom (the bulk cohesive energy of Co) when *x* → ∞.

A given number of Co atoms within a (3 × 3) or (4 × 4) surface unit cell can also be interpreted as a certain Co coverage. We can thereby convert the *x*-axis in Figure 3a to the Co coverage scale. For instance, a single Co adatom in the (3 × 3) unit cell corresponds to 0.05 ML of uniformly distributed Co adatoms on CeO_2_(111). It should be kept in mind that a distribution of particle sizes resulted in the experiments (cf., Figure 1). At a coverage comparable to 0.05 ML, therefore, NPs consisting of more than 10 or even 20 Co atoms can be readily found.

**Figure 5:**
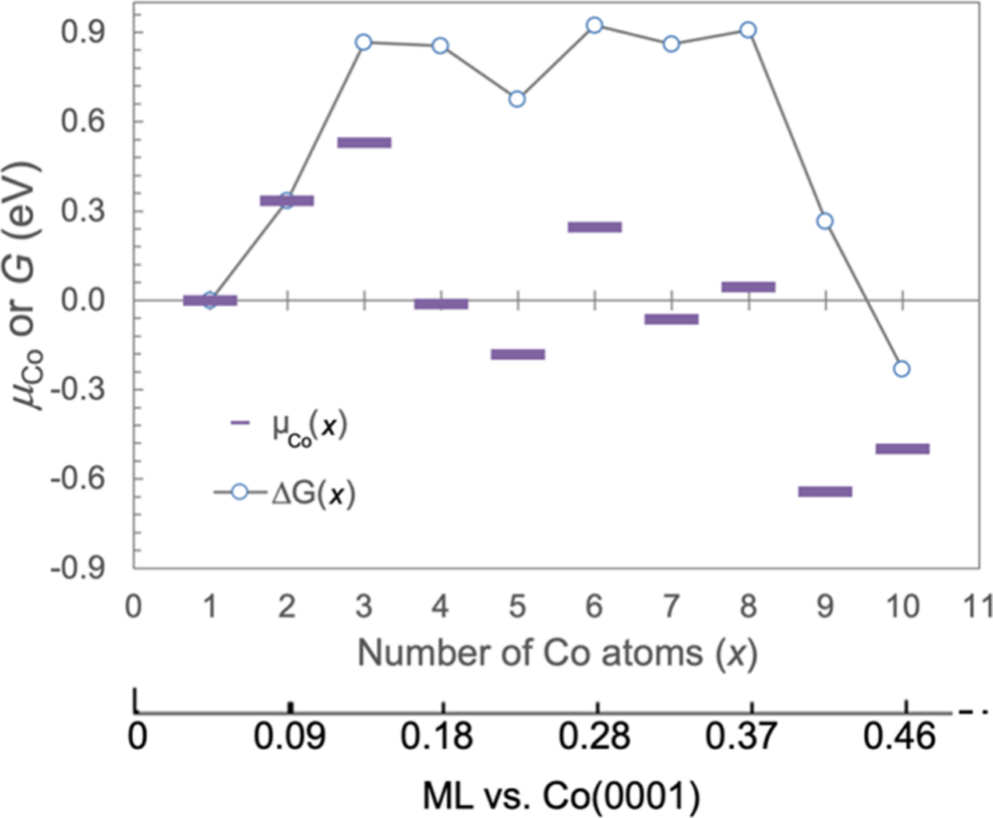
Calculated chemical potential of a Co atom in the ME Co NPs, defined as μ_Co_(*x*) = *E*_Co_*x*__ – *E*_Co_*x*-1__ – *E*_Co_1__, and change in free energy due to agglomeration, defined as Δ*G*(*x*) = *E*_Co_*x*__– *x*·*E*_Co_1__, as a function of number of atoms in Co NPs on CeO_2_(111). Δ*G*(*x*) is also equal to ∑_*i*_^*x*^μ_Co_(*i*).

Based on Figure 3, all NPs that lie above the Co adatom on the energy axis are less stable than it, while those that lie below it are more stable than it (see dashed line in Figure 3). Thus, a uniform 0.05 ML of Co adatoms prefers not to aggregate to form Co_4–9_, but increasing the Co coverage raises the chemical potential (μ) of a Co adatom (Figure 5), such that at ∼0.15 ML (3 adatoms per (3 × 3) surface unit cell), Co adatoms are no longer stable relative to any of the larger NPs. This suggests that there is a thermodynamic barrier to be overcome by Co adatoms to form larger, more stable NPs, i.e., to nucleate, which amounts to a total of Δ*G* = ∼ 0.9 eV (Figure 5). This does not account for additional barrier that may exist for, e.g., diffusion or geometry rearrangement. After nucleation, thermodynamics favors further clustering of Co adatoms into increasingly large particles and ultimately bulk Co metal. The process may fail to go to completion and is instead suspended at some intermediate particles size due to insufficient diffusion kinetics for adatoms and NPs.

As mentioned above, previous^53,54^ and the present XPS studies have indicated that Co adsorption reduces CeO_2_(111). Furthermore, our simulations reveal that Co NPs can also reduce CeO_2_(111) by extracting an O atom from the oxide surface, leaving an oxygen vacancy below. For *x* = 6–8, such Co_*x*_-O NP and vacancy combinations (blue circles, Figure 3a) have lower Δ*Ẽ* than the Co-only NPs reported above. For larger *x*, energetically competitive Co_*x*_-O NPs have also been found. The formation of such Co_*x*_-O NPs has activation barriers that are yet to be determined. Transfer of oxygen from ceria to Co NPs supported on it has been suggested by Martono and Vohs to occur below 400 K in a study of ethanol dehydrogenation on Co/CeO_2_/YSZ(100) model catalysts.^18^ Vári et al. reported LEIS evidence that oxygen diffuses from ceria to Co when 0.2 ML of Co is deposited on CeO_2_(111) at as low as 300 K.^20^ There is no direct information regarding the nature of this transfer, whether it involves reverse oxygen spillover or a type of SMSI where Co NPs are encapsulated by ceria, which is likely to occur when heating metal particles over reducible supports to high temperatures. This oxygen transfer is likely limited to NPs of Co.^18^ Indeed, the reduction of bulk ceria by Co metal according to the following formula has Δ*H* = +143 kJ/mol:^60,61^
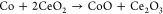


### Simulated STM and Dimensions of Co NPs

**Figure 6:**
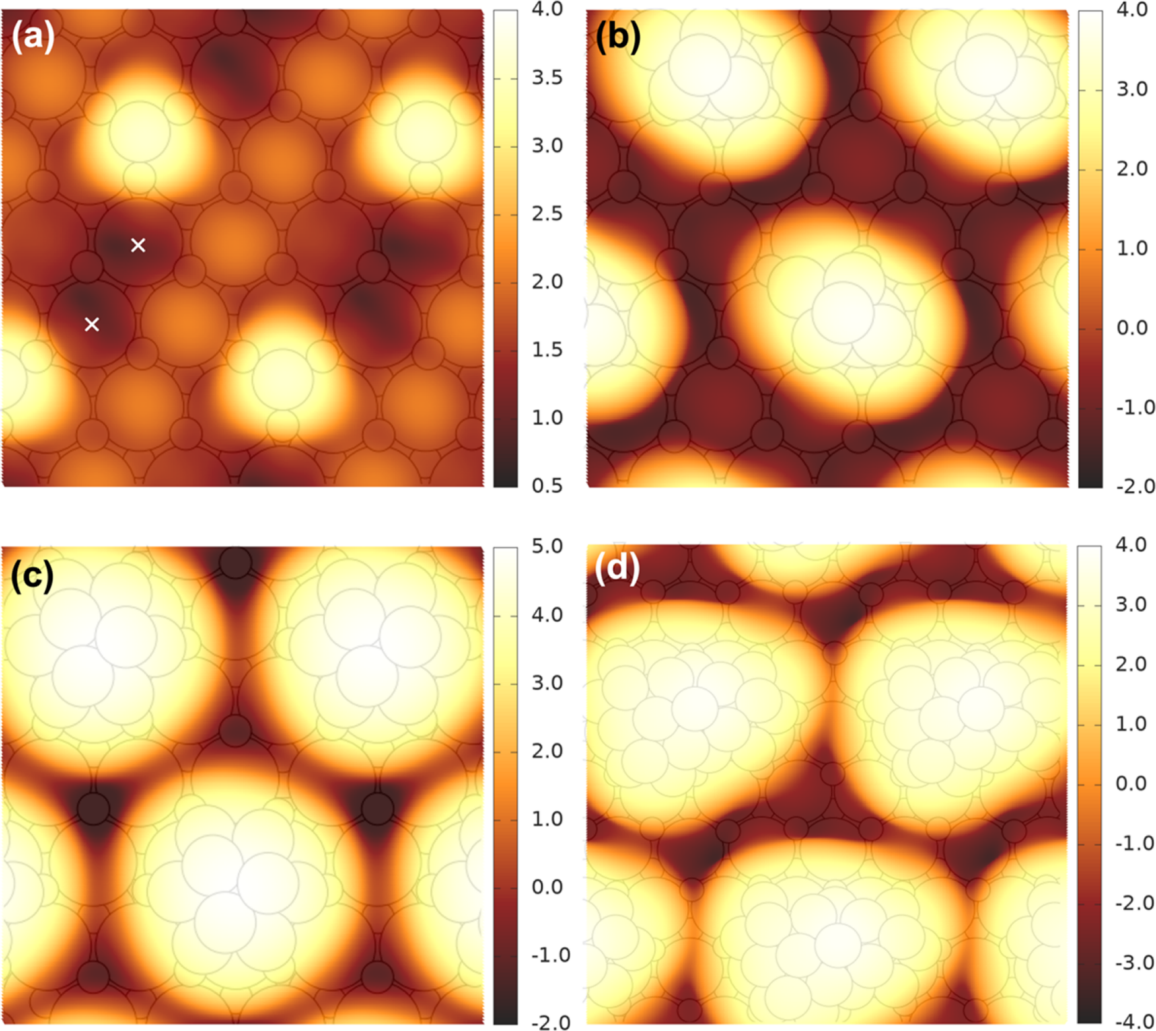
Simulated STM images of minimum-energy (a) Co_1_, (b) Co_5_, (c) Co_10_, and (d) Co_23_ on CeO_2_(111) in the Tersoff-Hamann formalism at an iso-density contour of 1.0 × 10^–2^*e*/Å^3^ and a sample voltage of +3.0 V. The relative height scale is indicated in Å for the tip. The images are overlaid on the corresponding surface structures to illustrate the position of the atoms. “×” in (a) marks the positions of the two Ce^3+^ species that occur with Co_1_ (repeating positions not marked).

STM images are simulated for Co_1_, Co_5_, Co_10_, and Co_23_ via the Tersoff-Hamann formalism^62,63^ at an iso-density surface of 1.0 × 10^–2^*e*/Å^3^ and a sample voltage of +3.0 V (Figure 6), in keeping with our previous STM study that was performed in a constant current mode at +3.0 ∼ + 3.5 V.^22^ Complex tip effects are beyond the scope of this work. In STM measurements, the precise geometry and electronic structure of the tip are generally unknown. The Tershoff-Hamann formalism invokes a simple *s* function for the tip with the interpretative advantage that the simulated STM images reflect the electronic structure of the surface alone. In the constant current mode, the STM tip is taken as moving along an iso-density contour derived from the partial charge density arising from the states in the given energy range positive of the Fermi level. The tunneling current is thus proportional to the local density of states at the position of the tip.

All four Co NPs are seen as bright features in simulated STM at a positive bias, which agrees with the appearance of Co NPs seen in the STM experiment (Figure 1a). The simulated STM for Co_1_ most clearly reveals the electronic structure of the ceria surface beneath. The regular arrays of (less) bright features in Figure 6a correspond to Ce^4+^ cations^64,65^ in the second atomic layer, which is also visible to some extent in Figure 6b,c. The metal cations and not the oxygen atoms are visible because the positive tip potential samples the empty metal states (Co 3d and Ce 4f) above the Fermi level. The “×” marks the positions of the two Ce^3+^ centers that occur with the Co adatom, which appear as dark spots because the Ce 4f states are occupied and do not accept tunneling current in a positively biased image.

**Figure 7:**
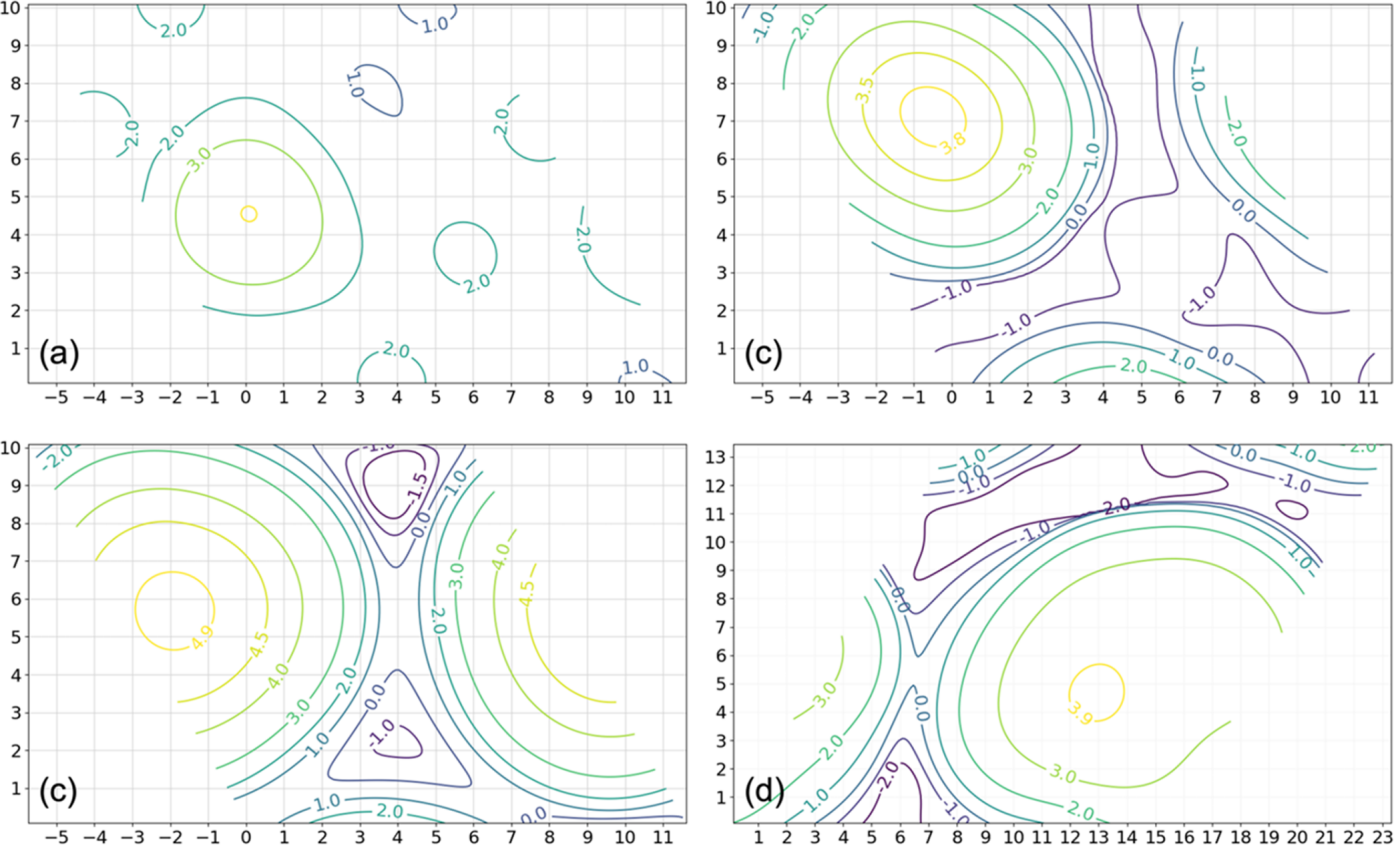
Relative height contours (values in Å) corresponding to the simulated STM images of (a) Co_1_, (b) Co_5_, (c) Co_10_, and (d) Co_23_ on CeO_2_(111) as shown in Figure 6. The *x*, *y* axes are also in Å. The maximum height in each panel is (a) 3.61 3.88, (b) 3.88, (c) 4.99, and (d) 3.98 Å.

The apparent height and diameter of each of the four Co NPs are analyzed based on the height contours of the iso-density surface in simulated STM (Figure 7). The results are reported together with the line profile analysis presented above (Figure 1d,e). The apparent diameter of an NP is taken to be between where the gradient d*d*/d*h* along the maximum lateral dimension exceeds −1.0, i.e., where the apparent height begins to decrease rapidly. The height difference between this point and the maximum height is taken to be the apparent height of the NP. This definition fails for Co_1_ because such a sufficiently small value d*d*/d*h* is not found on the surface. For Co_1_, therefore, we report the difference between the maximum and minimum heights as the apparent height of Co_1_ in Figure 1b. For comparison, we also plot in Figure 1d the widest distance between any pair of peripheral O_latt_ atoms bonded to each Co NP. This geometric measure turns out to be smaller than the apparent diameters in the simulated STM, which are in turn smaller than the prevailing diameters measured by line profile analysis for NPs of the same sizes. The apparent heights in the simulated STM are also greater than the line profile results, leading to aspect ratios (*h*:*d*) that are several times larger than what is obtained based on the average particle height and diameter values measured using the STM line profile method reported above, i.e., 1.1/16.1 Å = 0.07: 0.29 for Co_5_, 0.26 for Co_10_, and 0.24 for Co_23_. As can be seen in Figure 1d,e, in terms of percentage, a greater discrepancy between the measured and theoretical dimensions occurs with the apparent height. The simulated apparent height can be reduced somewhat by choosing a smaller iso-density contour. For instance, an iso-density value of 5.0 × 10^–3^*e*/Å^3^ yields a slightly lower ratio of 0.20 for Co_23_. This discrepancy may be due to tip convolution effects or indicate particle geometries that are not well represented by the ME structures reported here, which could occur due, e.g., to kinetics at room temperature that limit the transition of 2D to 3D structures. A detailed investigation of the structure and nature of individual Co NPs will need to be performed to resolve this discrepancy.

### Oxidation State of Co

We previously demonstrated using XPS that a decrease in the peak intensity of Ce^4+^ and a concomitant increase in the peak intensity of Ce^3+^ occurred when Co atoms were deposited on a CeO_1.97_(111) film, and that only Co^2+^ species were detected at low Co coverage while a growing fraction of Co was Co^0^ species with increasing Co coverage.^22^ Our theoretical results give us an alternative way to probe the oxidation state of Co upon deposition on CeO_2_(111), as a function of particle size. We perform Bader charge analysis using the code of Henkelman and co-workers^66,67^ to examine the oxidation state of Co atoms.

For Co_1_ (i.e., a Co adatom; Figure 4a), two Ce^3+^ atoms are identified on the surface (see Figure 6a). A Ce^3+^ atom is identified as one that gains approximately the same amount of charge as appears on each of two Ce atoms in a clean CeO_2_(111) surface upon the creation of a surface oxygen vacancy, ca. 9.88 *e* vs 9.62 *e* in a stoichiometric surface.^68^ Thus, for *x* = 1–3, each Co adatom loses 1.0–1.1 *e* in terms of Bader charge and is reckoned to be oxidized by the surface to Co^2+^. This is different from a Co atom substitutionally doped into CeO_2_, where it becomes Co^3+^.^69,70^ For *x* > 3, where Co atoms preferentially aggregate, two types of Co atoms are identified: All Co atoms that are in direct contact with the ceria surface are partially oxidized, but all those that are separated from the surface by the bottommost layer of Co are effectively screened and are neutral (Co^0^; with no or less than 1% excess charge). The only exposed part of the Co_*x*_ NPs that is positively charged is the peripheral Co atoms in contact with the oxide surface and not the entire exterior. If the latter were true, Co_*x*_ NPs would experience widespread repulsion among one another and cause the surface to be notably positively charged, which represents an unstable surface state.

**Figure 8:**
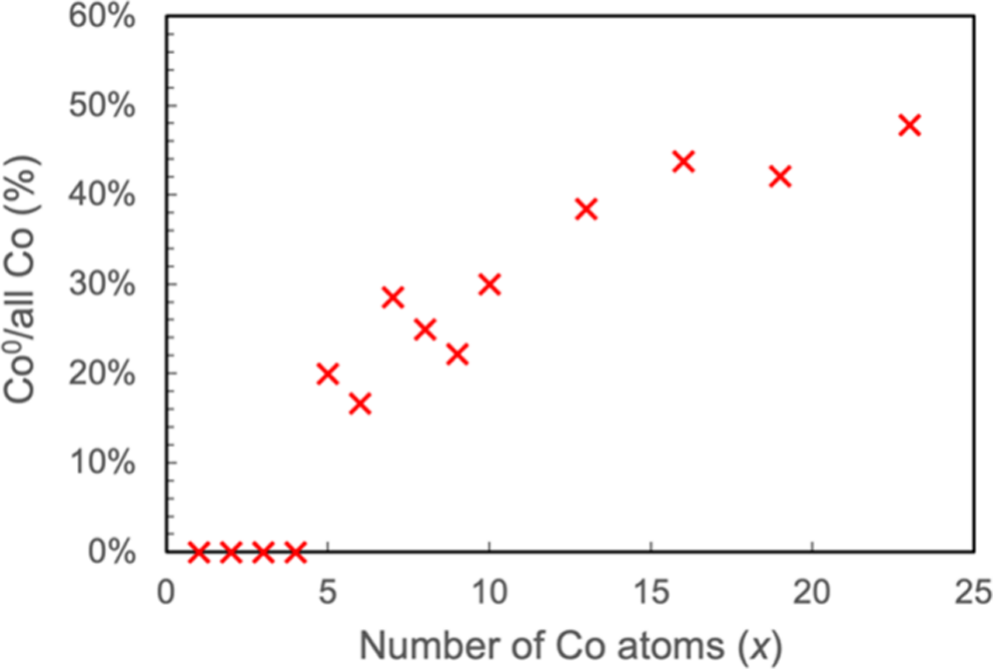
Percentage of Co atoms in Co NPs on CeO_2_(111) that is Co^0^ as deduced from Bader charge analysis, plotted against the number of Co atoms in Co NPs on CeO_2_(111).

**Figure 9:**
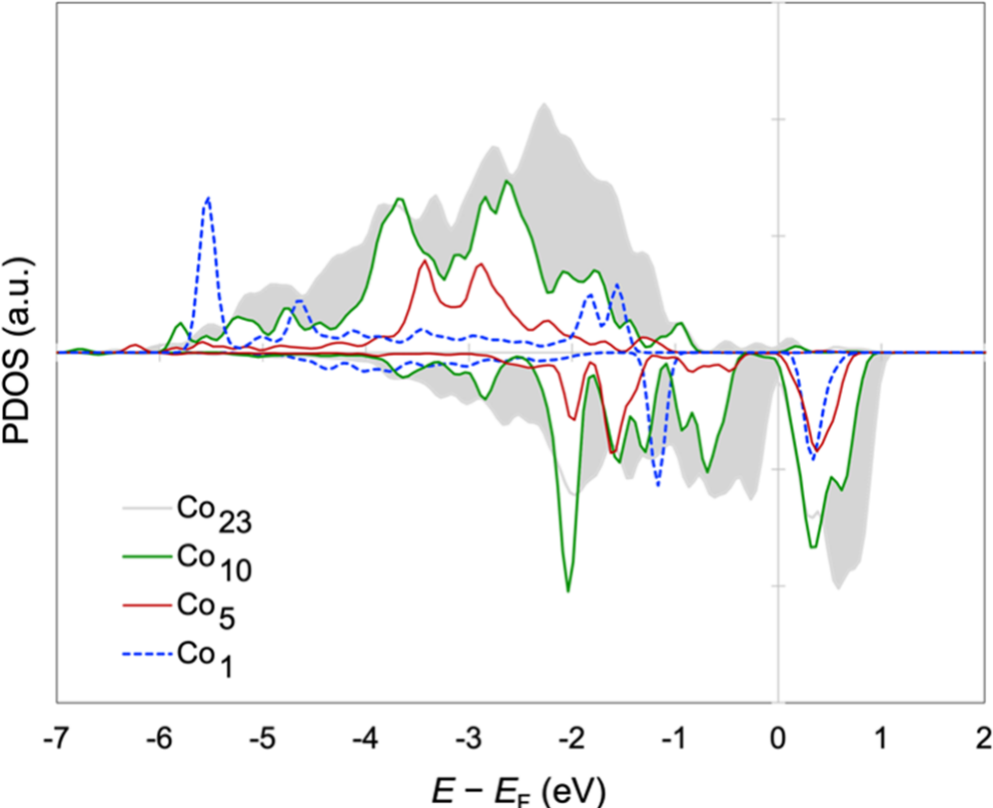
Projected density of states (PDOS) of Co *d* states in ME Co_1_, Co_5_, Co_10_, and Co_23_ on CeO_2_(111). Except for Co_1_, only the *d* states of the Co^0^ atoms are included in the plot.

The fraction of Co atoms in an NP that is Co^0^ increases with the particle size *x* (Figure 8). The Co^0^ species in these NPs may or may not be metallic, despite being neutral. The project *d* density of states for the neutral Co atoms in several NPs is plotted in Figure 9. Discrete states can be identified in, e.g., Co_1_, Co_5_, and Co_10_. The *d* states of the Co^0^ atoms in Co_23_ do appear band-like whether individually (not shown) or collectively. The spin splitting and significant DOS above the Fermi level (*E*_F_) in Co_23_ are evident in Figure 9.

The oxidic Co atoms in the ME NPs of *x* > 3 are not Co^2+^. They have lost on average 0.30–0.33 *e* each, which is approximately 1/3 of what is lost by the Co^2+^ species and corresponds to a formal charge of +0.6. We designate these partially oxidized Co atoms as Co^δ+^, with δ < 1. Thus, these Co NPs on CeO_2_(111) exhibit site ensembles (not considering site geometries) including Co^0^-Co^0^, Co^0^-Co^δ+^, and Co^δ+^-Co^δ+^, whose relative abundance changes with *x*. Adjacent Co atoms with distinct oxidation states may enable pathways not feasible on metallic Co, e.g., locally altered adsorption energetics that affect selectivity or break scaling relations. In Co_*x*_-O NPs, those Co atoms bonded to the O atom that is extracted from the lattice are also Co^δ+^ species.

The present and previous XPS studies of Co deposited on CeO_2_(111) at ambient temperature^20,22,68^ have detected a somewhat perturbed Co^2+^ signal at low coverages and then an increasingly intense Co^0^ signal when coverage is increased. The picture revealed by XPS is at odds with what the calculations suggest, i.e., only Co adatoms are Co^2+^ while a distribution of particle size exists at as low as 0.02 ML, in which the vast majority of Co atoms exists as either Co^δ+^ or Co^0^. The former has no corresponding bulk counterpart, and it is unknown whether it shows up as Co^2+^ in XPS. The latter species may not be the same as metallic Co at low coverage but is certainly expected to become metallic Co as coverage increases. As alluded to earlier, as the particle size decreases, final state effects tend to dominate that can cause broadening and deviations of the signals based on the bulk references. Further investigation will be needed to determine whether the theoretically computed oxidation states of the Co NPs, particularly the presence of Co^δ+^, are consistent with the XPS results.

## Conclusions

The nature of Co NPs formed on CeO_2_(111) has been analyzed in detail using STM and computational modeling based on DFT calculations. This work focuses on NPs on terrace sites. STM indicates that even at a very low coverage of ca. 0.02 ML, a wide distribution of particle sizes is found including those that contain more than 20 Co atoms. Line profile analysis suggests flat appearances for the Co NPs, with the average diameter increasing much faster than the average height. Computationally, Co NPs containing *x* = 1–10, 13, 16, 19, and 23 Co atoms adsorbed on CeO_2_(111) have been globally optimized using the minima hopping algorithm combined with DFT calculations. Many of the minimum-energy configurations exhibit some degree of symmetry. The results show individual Co adatoms at low coverage, ca. 0.05 ML to be more stable than NPs up to *x* = 10. No Co dimer or trimer configuration is found to be more stable than two or three separate Co adatoms in the given (3 × 3) surface unit cell. Simulated STM for several of the minimum-energy Co NPs (Co_1, 5, 10, and 23_) show them to be bright features on the ceria surface, albeit with aspect ratios that are considerably larger than the line profile measurements. This may be due to an electronic effect or may reflect geometries that deviate from the theoretically determined configurations.

Our DFT results indicate individual Co adatoms to be Co^2+^ species, while Co NPs contain partially oxidized Co^δ+^ and neutral Co^0^ species, but no Co^2+^. All interfacial Co atoms (including those located at the periphery) in each NP are found to be Co^δ+^, while any remaining Co atoms, being screened by the Co^δ+^ atoms, are neutral but not necessarily metallic in terms of electronic structure depending on the particle size. Given the distribution of particle size that exists even at low Co coverages, a mixture of Co^0^, Co^δ+^, and Co^2+^ species may coexist upon Co deposition on CeO_2_(111), with the fraction of Co^0^ increasing with Co coverage and therefore with distribution toward larger particle sizes. A potential discrepancy in the interpretation of the chemical state of Co between the experimental XPS data and DFT results also remains to be further investigated.
